# Bioaccumulation and Biomagnification of Polychlorinated Biphenyls and Dichlorodiphenyltrichloroethane in Biota from Qilianyu Island, South China Sea

**DOI:** 10.3390/toxics10060324

**Published:** 2022-06-14

**Authors:** Qingling Wang, Chenmin Xie, Chuyue Long, Weiyan Yang, Yan Wang, Weihai Xu, Li Zhang, Yuxin Sun

**Affiliations:** 1 Research Center of Harmful Algae and Marine Biology, Jinan University, Guangzhou 510632, China; ql_wang1997@163.com (Q.W.); 202033321007@stu2020.jnu.edu.cn (W.Y.); 2 CAS Key Laboratory of Tropical Marine Bio-resources and Ecology, Guangdong Provincial Key Laboratory of Applied Marine Biology, South China Sea Institute of Oceanology, Chinese Academy of Sciences, Guangzhou 510301, China; xiechenmin19@mails.ucas.ac.cn (C.X.); longchuyue20@mails.ucas.ac.cn (C.L.); zhangli@scsio.ac.cn (L.Z.); sunyx@scsio.ac.cn (Y.S.); 3 Sanya Institute of Oceanology, South China Sea Institute of Oceanology, Chinese Academy of Sciences, Sanya 572000, China; whxu@scsio.ac.cn

**Keywords:** PCBs, DDTs, bioaccumulation, biomagnification, risk assessment, South China Sea

## Abstract

Six biota species were collected from Qilianyu Island, South China Sea to determine the bioaccumulation and biomagnification of polychlorinated biphenyls (PCBs) and dichlorodiphenyltrichloroethane and its metabolites (DDTs). Concentrations of ΣPCBs and ΣDDTs in biota from Qilianyu Island ranged from 6.88 to 519.1 ng/g lipid weight (lw) and 7.0 to 19,413 ng/g lw, respectively. Significant differences for PCBs and DDTs concentrations were found among the six biota species from Qilianyu Island. The levels of PCBs and DDTs in intermediate egret were significantly higher than the other five biota species, which can be attributed to their different feeding and living habits. Significantly negative relationships between concentrations of PCBs and DDTs and δ13C values in the six biota species confirmed that dietary source is an important factor to determine the levels of PCBs and DDTs in biota species. ΣPCBs, ΣDDTs, PCB 28/31, PCB 52, and *p,p′*-DDE were biomagnified in the biota species from Qilianyu Island, and native species are suitable for studying the biomagnification of the contaminants. The toxic equivalent concentrations in birds from Qilianyu Island were significantly and positively correlated with PCBs concentrations, indicating that high concentrations of non- and mono-*ortho*-PCB congeners may induce adverse effects on bird species.

## 1. Introduction

Polychlorinated biphenyls (PCBs) and dichlorodiphenyltrichloroethane (DDT) are two typical types of chlorinated persistent organic pollutants (POPs) and have been of great concern for several decades due to their persistence, toxicity, bioaccumulation and long-range transport. PCBs were mainly used as dielectric and hydraulic fluids in industrial products such as transformers, capacitors, and electric motors from the 1930s to 1970s [1]. DDTs were widely used as an agricultural insecticide in the 1940s and 1950s, and the use of DDTs was prohibited in the early 1970s [2]. It is estimated that China has produced about 10,000 tons of PCBs and 400,000 tons of DDTs [3,4]. Due to the increasing concern about the adverse effects of PCBs and DDTs on wildlife and human health, the production of PCBs and DDTs was banned in the late 1970s and in the mid to late 1980s, respectively. PCBs and DDTs have been added in the initial list of 12 POPs under the Stockholm Convention in May 2001. The production and use of DDTs was prohibited in 2009. However, DDTs are still used in some countries to control certain insects and as antifouling paints for fishing boats [5]. PCBs and DDTs are still slowly and continuously released from old equipment and waste dumps into the environment, which can expose wild animals and humans [6]. PCBs and DDTs can be bioconcentrated into higher trophic levels of biota through the food chain and eventually absorbed by humans, adversely affecting human health [7].

The South China Sea (SCS) is one of the richest areas of marine biodiversity in the world, surrounded by several developing countries that are considered as potential sources of POPs [8,9]. POPs from these countries may enter the SCS through surface runoff, rain wash, and atmospheric deposition. The Qilianyu Island is a typical coral reef ecosystem of the SCS with abundant natural resources and various species. POPs in marine biota from the Qilianyu Island should deserve our attention because of not only their potential ecological impacts but also the public concerns for seafood safety. To date, little information is available about PCBs and DDTs in biota from coral reef ecosystem of the SCS [10,11]. Therefore, bioaccumulation and biomagnification of PCBs and DDTs in biota from the SCS is urgently needed.

In this study, six biota species, including two birds and four fishes, were collected from Qilianyu Island, SCS and analyzed for PCBs and DDTs. The main objective of this study was to investigate the bioaccumulation and biomagnification of PCBs and DDTs in biota from Qilianyu Island. The ecological risks of PCBs and DDTs to biota species were also evaluated.

## 2. Materials and Methods

### 2.1. Sample Collection

A total of six biota species, including intermediate egret (*Ardea intermedia*, *n* = 6), red-footed booby (*Sula sula*, *n* = 5), peacock grouper (*Cephalopholis argus*, *n* = 6), goldspot seabream (*Gnathodentex aureolineatus*, *n* = 4), Japan surgeonfish (*Acanthurus japonicus*, *n* = 4), and striated surgeonfish (*Ctenochaetus striatus*, *n* = 6), were collected from Qilianyu Islands, SCS. Intermediate egret is a migratory bird, and the other five species are native. Fishes were collected by fishing nets. Birds were found dead or stranded on the beaches of Islands. All biota samples were immediately transported to the laboratory and stored at −20 °C until chemical analysis.

### 2.2. Sample Extraction and Analysis

Muscles were excised from each biota sample and then freeze-dried and ground. Approximately 1 g of muscle samples were spiked with surrogate standards (PCB 30, 65 and 204) and then treated by microwave-assisted extraction with 30 mL acetone/hexane (*v*/*v* = 1:1) for 45 min. The extraction solutions were concentrated and converted to hexane. A total of 1 mL of the extract was used for the gravimetric determination of lipid content. Then, 9 mL of the extract was used for PCBs and DDTs analysis, added into concentrated sulfuric acid to remove lipid, and extracted with hexane. The extraction solutions were cleaned up on a multilayer column filled with 1 cm anhydrous sodium sulfate, 8 cm acidified silica, and 8 cm neutral silica from top to bottom. The extract was eluted with 30 mL hexane/dichloromethane (*v*/*v* = 1:1), and the eluate was concentrated to near dryness under a gentle nitrogen flow and reconstituted in 100 μL of hexane. Internal standards (PCB 24, 82 and 198) were spiked before instrumental analysis.

Thirty-four PCB congeners (PCB 28/31, 52, 66, 74, 77, 99, 105, 115/87, 118, 126, 128, 130, 138, 146, 149/139, 153, 156, 158, 164/163, 167,171, 172, 175, 180, 183, 187, 190, 191, 194, 195, 203, 205, 206, 209), DDT (*p,p*′-DDT), and its metabolites (*p,p*′-DDM, *p,p*′-DDMU, *p,p*′-DDE, *o,p*′-DDE, *o,p*′-DDD) were analyzed by an Agilent 7890 gas chromatograph coupled with an Agilent 5975B mass spectrometer using electron impact in the selective ion-monitoring mode and separated by a DB-5MS (60 m × 0.25 mm × 0.25 μm, J&W Scientific, Agilent Tech, USA) capillary column. The heating program was as follows: the initial oven temperature was set at 120 °C, then increased to 180 °C at a rate of 5 °C/min, further increased to 240 °C at a rate of 1 °C/min, and finally raised to 290 °C at a rate of 6 °C/min (held for 17 min). Then, 1 μL of sample was injected in the pulsed splitless mode. The monitored and quantitative ions of PCBs and DDTs were given elsewhere [12].

### 2.3. Quality Assurance and Quality Control (QA/QC)

A procedural blank was performed in each batch of the sample analysis. Triplicate spiked blanks (PCB 28, 52, 60, 66, 87, 99, 101, 107, 110, 118, 128, 136, 138, 141, 153, 164, 170, 174, 180, 183, 187, and 209) and triplicate spiked matrices were passed through the whole analytical procedure to ensure method QC. PCB 28 was detected in the procedural blanks with mean concentrations of 2.88 ng/mL and subtracted from the samples. The average recoveries of PCB congeners in the spiked blanks and matrices ranged from 106.5% to 129.4% and 109.6% to 124.1%, respectively. The relative standard deviations were less than 6%. The mean recoveries of surrogate standard in all samples were 70.1 ± 2.7% for PCB 30, 74.3 ± 2.8% for PCB 65, and 74.7 ± 2.2% for PCB 204, respectively. Method detection limits (MDLs) were defined as 3 times the signal-to-noise levels for the undetected compounds in blanks. MDLs were set as three times the standard deviation of the target value in blanks. Based on the average lipid weight (lw) of samples, the MDLs ranged from 0.15 to 1.10 ng/g lw for PCBs and 0.19 to 6.76 ng/g lw for DDTs, respectively.

### 2.4. Stable Isotope Analysis

The samples for ẟ^13^C and ẟ^15^N analysis were freeze-dried and ground into powder. A roughly 0.4 mg sample was placed into a tin vessel and analyzed for stable carbon and nitrogen isotopes by a Flash 2000 series elemental analyzer coupled to a Delta V isotope ratio mass spectrometer (Thermo Scientific, Germany). The analytical precision was less than 0.2‰. The abundance of stable isotope values is calculated by the following formula:

δX = (Rsample/Rstandard − 1) × 1000

where δX is δ^13^C or δ^15^N, and R is the corresponding ratio of ^13^C/^12^C or ^15^N/^14^N.

### 2.5. Data Analysis

Concentrations were expressed as a lipid weight (lw) basis. Statistical analysis was performed with SPSS 22 (SPSS Inc., Illinois, USA). The level of significance was set as *p* < 0.05. Concentrations were not normally distributed and log_10_ transformed before statistical analysis. Independent-*sample t*-test was used to compare the differences of δ^13^C and δ^15^N values between birds and fishes. One-way analysis of variance (ANOVA) was used to analyze the differences in concentrations of PCBs and DDTs among the six biota species. Linear regression analysis was used to investigate the correlations between concentrations and δ^13^C values, concentrations and δ^15^N values, PCBs, and TEQ concentrations in biota.

## 3. Results and Discussion

### 3.1. Stable Isotope of δ^13^C or δ^15^N in Biota

**Figure 1:**
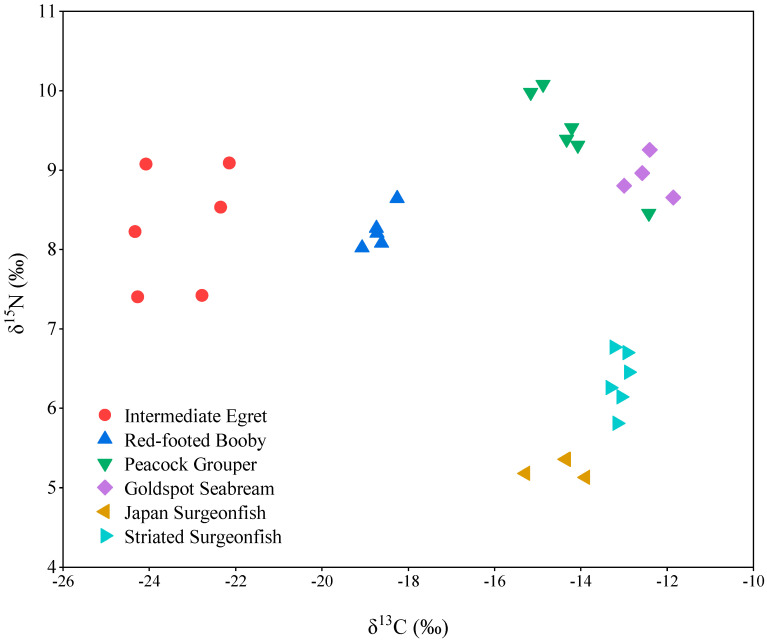
Stable isotope ratios of carbon and nitrogen in biota from Qilianyu Island.

δ^13^C and δ^15^N values are often used to infer the carbon source and trophic level in biota [13]. Significant differences for δ^13^C and δ^15^N values were observed in biota from Qilianyu Island (*p* < 0.001). The δ^13^C values increased the order of intermediate egret (−23.3 ± 0.41‰) < red-footed booby (−18.7 ± 0.13‰) < Japan surgeonfish (−14.5 ± 0.29‰) < peacock grouper (−14.2 ± 0.39‰) < striated surgeonfish (−13.1 ± 0.07‰) < goldspot seabream (−12.5 ± 0.24‰) (Figure 1). The δ^13^C values of the two bird species were significantly different from the four fish species. Intermediate egret is a migratory and wading bird, mainly feeding on fish, shrimp, frog, locust, aquatic and terrestrial insects, and small invertebrates. The red-footed booby is a resident bird, predominantly feeding on fish, squid, and crustacean and living on the islands [14]. The four fishes live in the sea and feed on marine organisms. Peacock grouper is a carnivorous fish, feeding on small fish and invertebrates. Goldspot seabream also is a carnivorous fish, feeding on polychaetes and shellfish. Japan surgeonfish and striated surgeonfish are herbivorous fish and mainly feed on algae. The results indicated that biota with a more marine feeding strategy have higher δ^13^C values. The δ^15^N values decreased in the order of peacock grouper (9.46 ± 0.24‰) > goldspot seabream (8.92 ± 0.13‰) > intermediate egret (8.29 ± 0.31‰) > red-footed booby (8.24 ± 0.11‰) > striated surgeonfish (6.36 ± 0.15‰) > Japan surgeonfish (5.26 ± 0.06‰). The δ^15^N values of two herbivorous fishes were significantly lower than those of two carnivorous fishes and two bird species, suggesting that the two fishes had a lower trophic level.

### 3.2. Bioaccumulation of PCBs and DDTs in Biota

**Figure 2:**
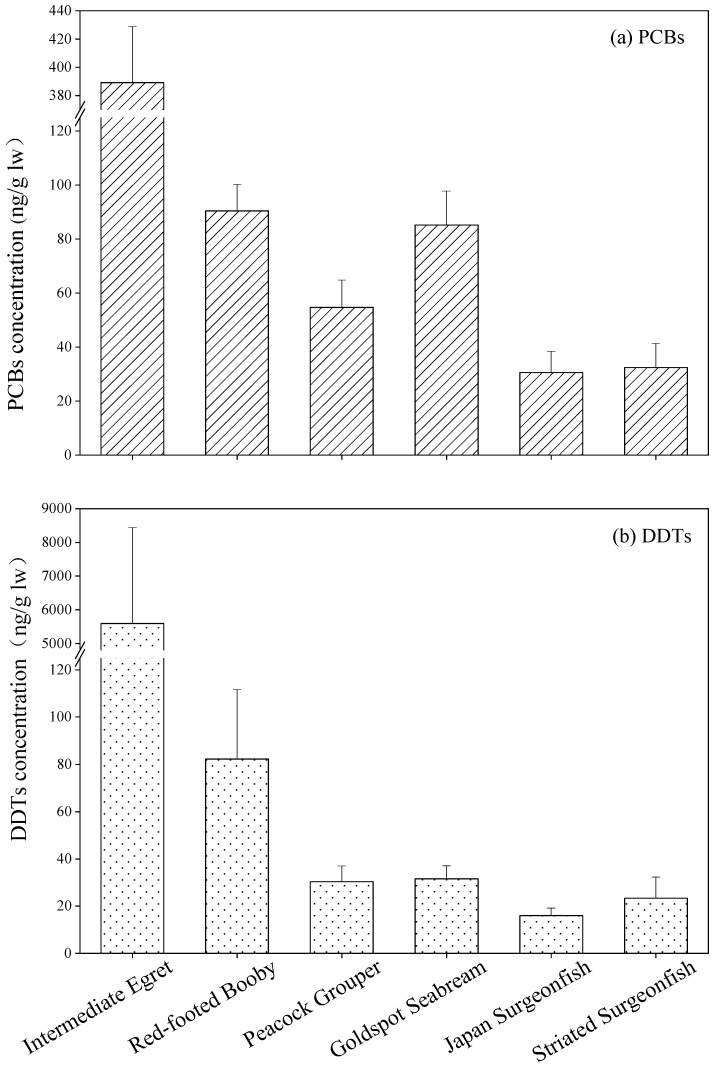
Concentrations of PCBs and DDTs (mean ± standard error) in biota from Qilianyu Island.

**Figure 3:**
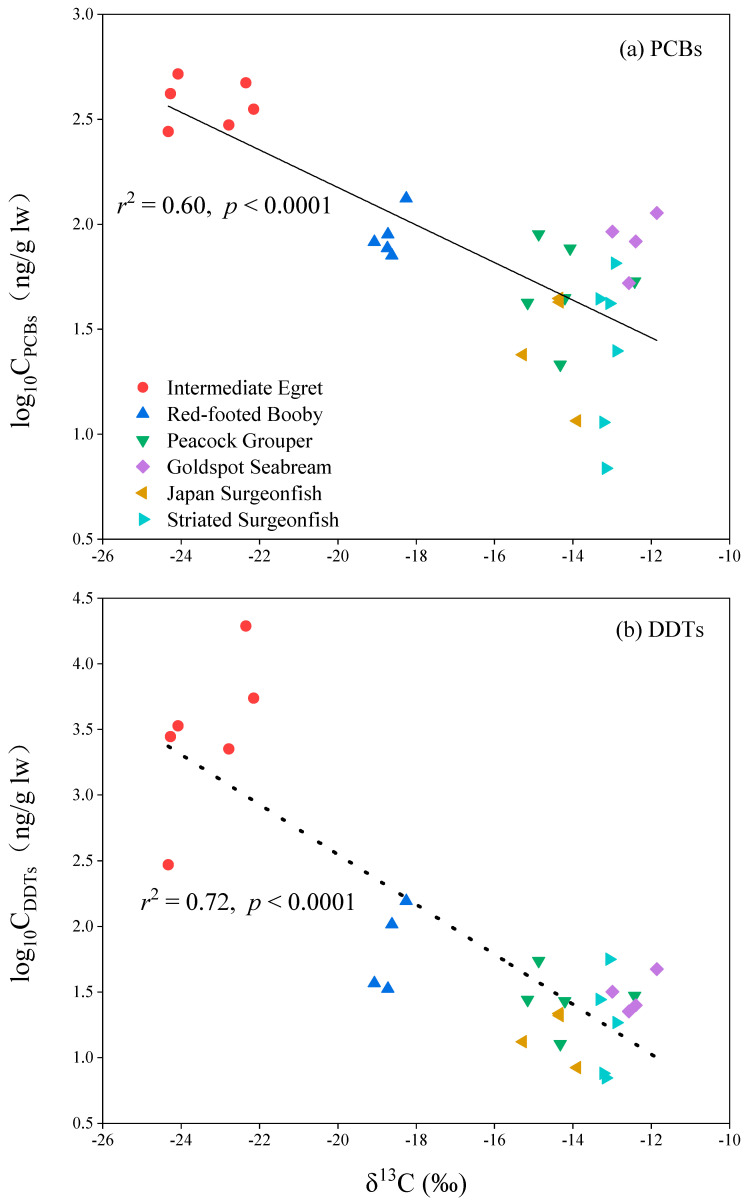
The relationship between concentrations of PCBs and DDTs and δ^13^C values.

Concentrations of PCBs in bird and fish species from Qilianyu Island ranged from 71.0–519.1 and 6.88–113.2 ng/g lw, respectively. The mean concentrations of PCBs in six biota species had the following order of intermediate egret (416.6 ± 43.6 ng/g lw) > red-footed booby (90.5 ± 11.0 ng/g lw) > goldspot seabream (85.1 ± 12.6 ng/g lw) > peacock grouper (54.7 ± 10.1 ng/g lw) > striated surgeonfish (32.4 ± 9.0 ng/g lw) > Japan surgeonfish (30.6 ± 7.8 ng/g lw) (Figure 2a). Significant differences for PCBs concentrations were found among the six biota species from Qilianyu Island (*F* = 47.3, *p* < 0.001). The levels of PCBs in intermediate egret were significantly greater than the other five biota species (*p* < 0.001), which can be explained by their different feeding and living habits. Intermediate egret is a migratory bird, and the other five biota species often live in the surrounding of Qilianyu Island. In addition, significantly negative relationships between PCBs concentrations and δ^13^C values were found among the six biota species (Figure 3a), further confirming that dietary source is an important factor to determine the concentrations of PCBs in biota species. Intermediate egret feed on marine and terrestrial biota, which may increase their exposure to PCBs from land. Therefore, relatively high concentrations of PCBs were found in the intermediate egret.

*o,p*′-DDE, *o,p*′-DDD, *p,p*′-DDMU, *p,p*′-DDE, and *p,p*′-DDT were detected in the intermediate egret. *o,p*′-DDE and *p,p*′-DDE were detected in the red-footed booby. For four fish species, only *p,p*′-DDE were detected. The concentrations of DDTs in biota from Qilianyu Island ranged from 7.0 to 19,413 ng/g lw. The highest mean concentrations of DDTs were found in the intermediate egret (5595 ± 2847 ng/g lw), followed by red-footed booby (82.3 ± 29.3 ng/g lw), goldspot seabream (31.6 ± 5.5 ng/g lw), peacock grouper (30.3 ± 6.8 ng/g lw), striated surgeonfish (23.3 ± 9.0 ng/g lw), and Japan surgeonfish (16.0 ± 3.2 ng/g lw) (Figure 2b). The concentrations of DDTs in intermediate egret were significantly greater than the other five biota species (*p* < 0.05), which were similar to PCBs distribution pattern. Significantly negative relationships between DDTs concentrations and δ^13^C values were seen among the six biota species (Figure 3b), indicating that dietary source is also an important factor to determine the concentrations of DDTs in biota species.

Penta-, hexa-, and hepta-PCBs were the main homologue profiles in the intermediate egret and red-footed booby, contributing more than 79% to the total PCBs. PCB 153, 180, 118, and 138 were the main PCB congeners in bird species (Figure S1, Supplementary Materials). Similar results were also found in bird species from the light-vented bulbul from Guangdong Province, South China [12]. Due to the presence of chlorine substitutions at *meta-para* positions on the phenyl rings, the four PCB congeners were slowly cleared in bird species [15]. For four fish species, only PCB 28/31, 52, 128, and 153 were detected and PCB 153 was the predominant congener (Figure S1).

*p,p*′-DDE was the most abundant compound in six biota species from Qilianyu Island and contributed more than 95% to the total DDTs (Figure S2). The ratio of (DDE + DDD)/DDTs is used to determine the source of DDT. If the ratio of (DDE + DDD)/DDTs is higher than 0.5, the source of DDTs come from historical use instead of fresh input [9]. In the present study, the ratios of (DDE + DDD)/DDTs in biota species ranged from 0.99 to 1.0, indicating that historical residue was the main source of DDTs in biota from Qilianyu Island. The highly bioaccumulative *p,p*′-DDE alongside the low concentrations and detectable frequency of *p,p*′-DDT (6.7%) also indicated that DDTs in biota from Qilianyu Island were derived from historical use rather than recent input.

### 3.3. Biomagnification of PCBs and DDTs

**Figure 4:**
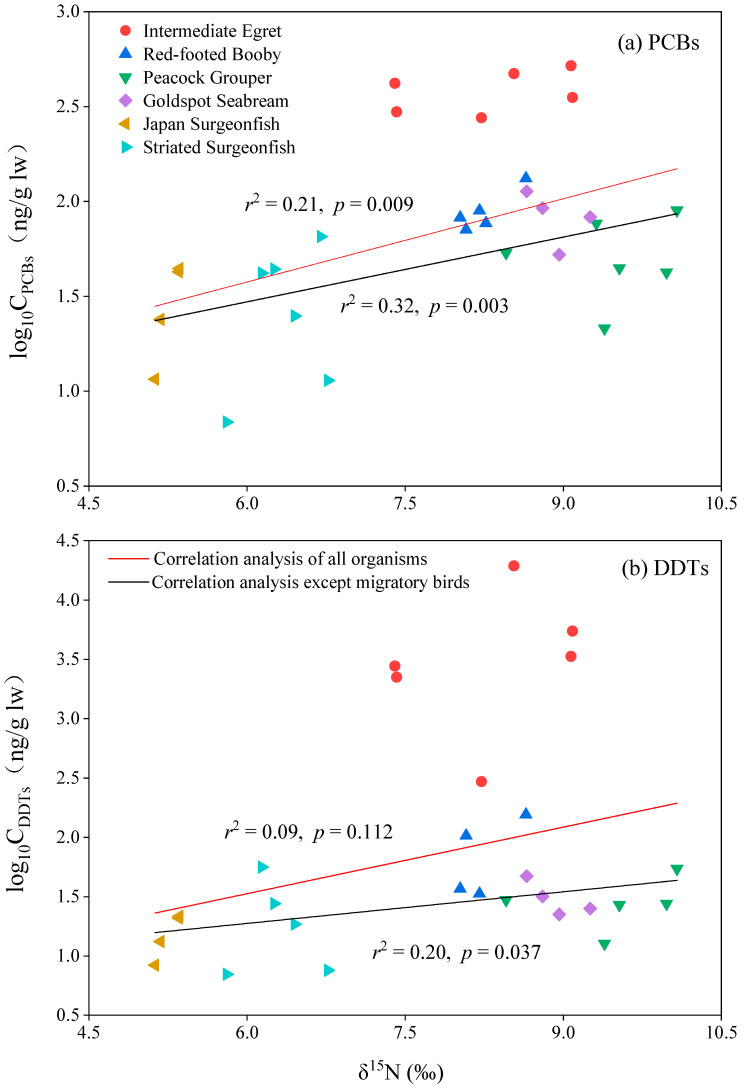
Relationships between concentrations of PCBs and DDTs and δ^15^N in biota species from Qilianyu Island.

The stable isotope of nitrogen was used to illustrate the trophic level of biota. The relationships between PCBs and DDTs concentrations and δ^15^N values were used to investigate the biomagnification of these contaminants. Concentrations of ΣPCBs and PCB 28/31 were positively and significantly correlated with δ^15^N values in all six biota species (Figure 4, Figure S3). Considering that intermediate egret is a migratory bird and temporarily lives in the surrounding environment of Qilianyu Island, we also investigated the relationship between PCBs and DDTs concentrations and δ^15^N values in five native species. After excluding the migratory bird intermediate egret, Concentrations of ΣPCBs, ΣDDTs, PCB 28/31, PCB 52, and *p,p*′-DDE had significant and positive correlations with δ^15^N values in the five native biota species (Figure 4, Figures S3 and S4). The results suggested that PCBs and DDTs can be biomagnified in the biota species from Qilianyu Island and native species are suitable for studying the biomagnification of the contaminants.

### 3.4. Risk Assessment

**Figure 5:**
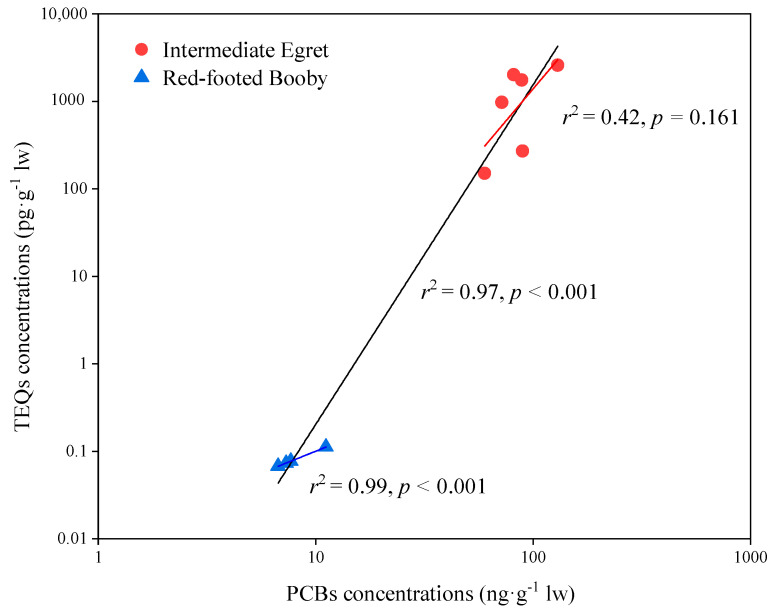
Correlations between PCBs and TEQ concentrations in birds from Qilianyu Island.

Non- and mono-*ortho*-PCB congeners can cause dioxin-like effects by binding affinity to the aryl hydrocarbon receptor. Therefore, toxic equivalency factors (TEFs) for wildlife risk assessment suggested by the World Health Organization (WHO) were used to evaluate the toxic equivalent (TEQ) concentrations of these PCBs [16]. Because of the low detection frequency of PCBs in fish, only the risk assessment of PCBs to birds was evaluated in this study. Non-*ortho*-PCB congeners (PCB 77 and 126) and mono-*ortho*-PCB congeners (PCB 105, 118, 156, and 167) were detected in bird samples. The concentrations of TEQs in intermediate egret and red-footed booby ranged from 270–2586 pg/g lw and 0.07–0.11 pg/g lw, respectively. The levels of TEQs in the intermediate egret were much higher than red-footed booby (*p* < 0.001). The TEQ concentrations in birds from Qilianyu Island were significantly and positively correlated with PCBs concentrations (Figure 5). A significant positive relationship between TEQ and PCB concentrations in bird species was also found in other studies [12,17], suggesting that high concentrations of non- and mono-*ortho*-PCB congeners may induce adverse effects on bird species.

It has been reported that bird populations had declined due to bioaccumulation of DDTs [18]. The concentrations of DDTs in the intermediate egret and red-footed booby ranged from 15.8–650.7 ng/g ww and 1.34–5.22 ng/g ww, respectively. The no-effect level of DDTs for little egret is 500 ng/g ww [19]. The concentrations of one intermediate egret (650.3 ng/g ww) exceeded the no-effect level for little egret. Both intermediate egret and little egret are Ardeidae birds. Thus, high concentrations of DDTs may cause reproductive effect on the intermediate egret. The concentrations of DDTs in fish from Qilianyu Island ranged from 0.10 to 0.36 ng/g ww, which were much lower than the no-observed-adverse-effect level for juvenile and adult fish (600 ng/g ww) and early-life-stage fish (700 ng/g ww) [20].

## 4. Conclusions

This study investigated the bioaccumulation and biomagnification of PCBs and DDTs in six biota species from Qilianyu Island, SCS. Biota with a more marine feeding strategy had higher δ^13^C values. The δ^15^N values of two herbivorous fishes were significantly lower than those of two carnivorous fishes and two bird species, suggesting that the two fishes had lower trophic level. Significant differences for PCBs and DDTs concentrations were found among the six biota species from Qilianyu Island. The levels of PCBs and DDTs in intermediate egret were significantly higher than the other five biota species, which can be attributed to their different feeding and living habits. Dietary source is an important factor to determine the concentrations of PCBs and DDTs in biota species. ΣPCBs, ΣDDTs, PCB 28/31, PCB 52, and *p,p*′-DDE were biomagnified in the biota species from Qilianyu Island, and native species are suitable for investigating the biomagnification of these contaminants. The high concentrations of non- and mono-*ortho*-PCB congeners may induce adverse effects on bird species, and the high concentrations of DDTs may cause reproductive effect on intermediate egret.

## Supplementary Materials

The following supporting information can be downloaded at: https://www.mdpi.com/article/10.3390/toxics10060324/s1, Figure S1: PCB congener profiles in biota from Qilianyu Island; Figure S2: Composition profiles of DDTs in biota from Qilianyu Island; Figure S3: Relationships between concentrations of PCB congeners and δ^15^N in biota species from Qilianyu Island; Figure S4: Relationships between concentrations of *p,p*′-DDE and δ^15^N in biota species from Qilianyu Island.
