# Supplementary material for: Bioaccumulation and Biomagnification of Polychlorinated Biphenyls and Dichlorodiphenyltrichloroethane in Biota from Qilianyu Island, South China Sea
Source: Toxics. 2022 Jun 14;10(6):324. doi: 10.3390/toxics10060324 (PMC9230657; doi:10.3390/toxics10060324)
Supplement: Supplementary file 1 [file toxics-10-00324-s001.zip › toxics-1743711-supplementary.pdf]

toxics

MDPI

# Supplementary Materials: Bioaccumulation and Biomagnification of Polychlorinated Biphenyls and Dichlorodiphenyltrichloroethane in Biota from Qilianyu Island, South China Sea

**Qingling Wang, Chenmin Xie, Chuyue Long, Weiyan Yang, Yan Wang, Weihai Xu, Li Zhang, and Yuxin Sun**

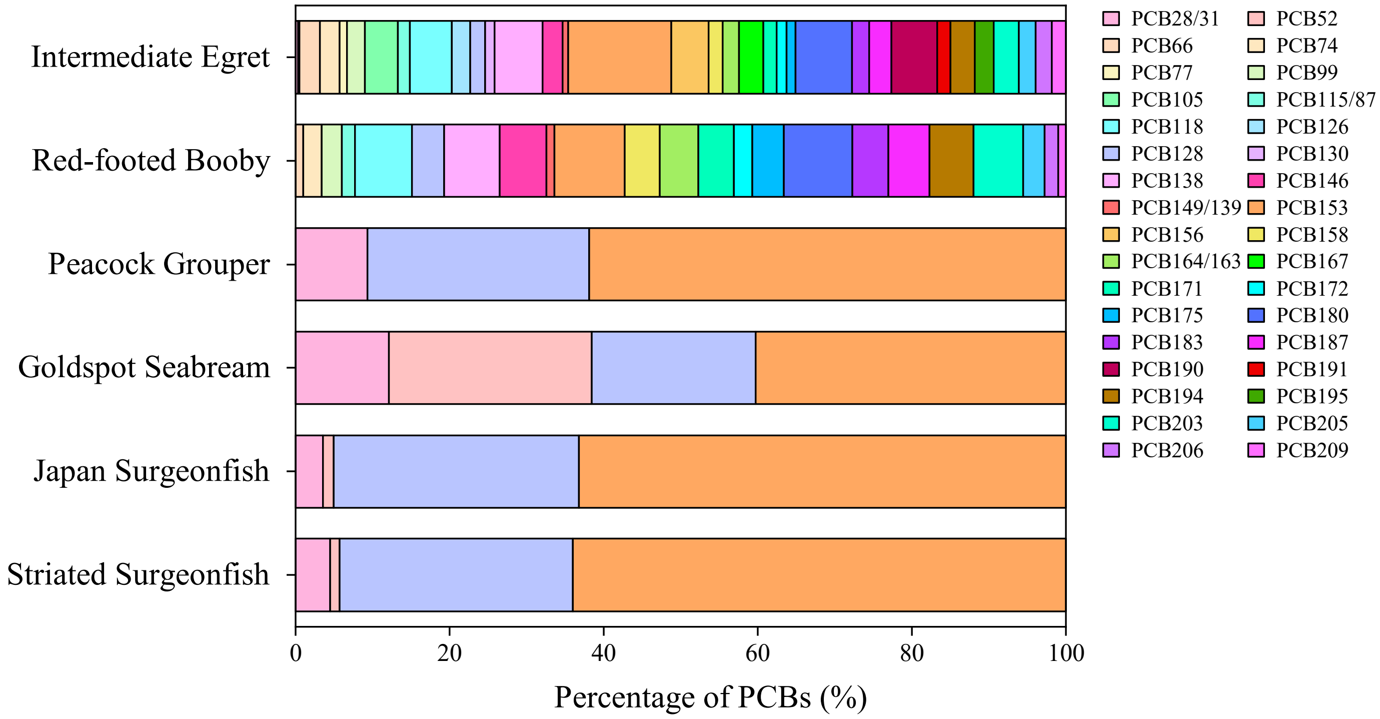


**Figure S1.** PCB congener profiles in biota from Qilianyu Island.

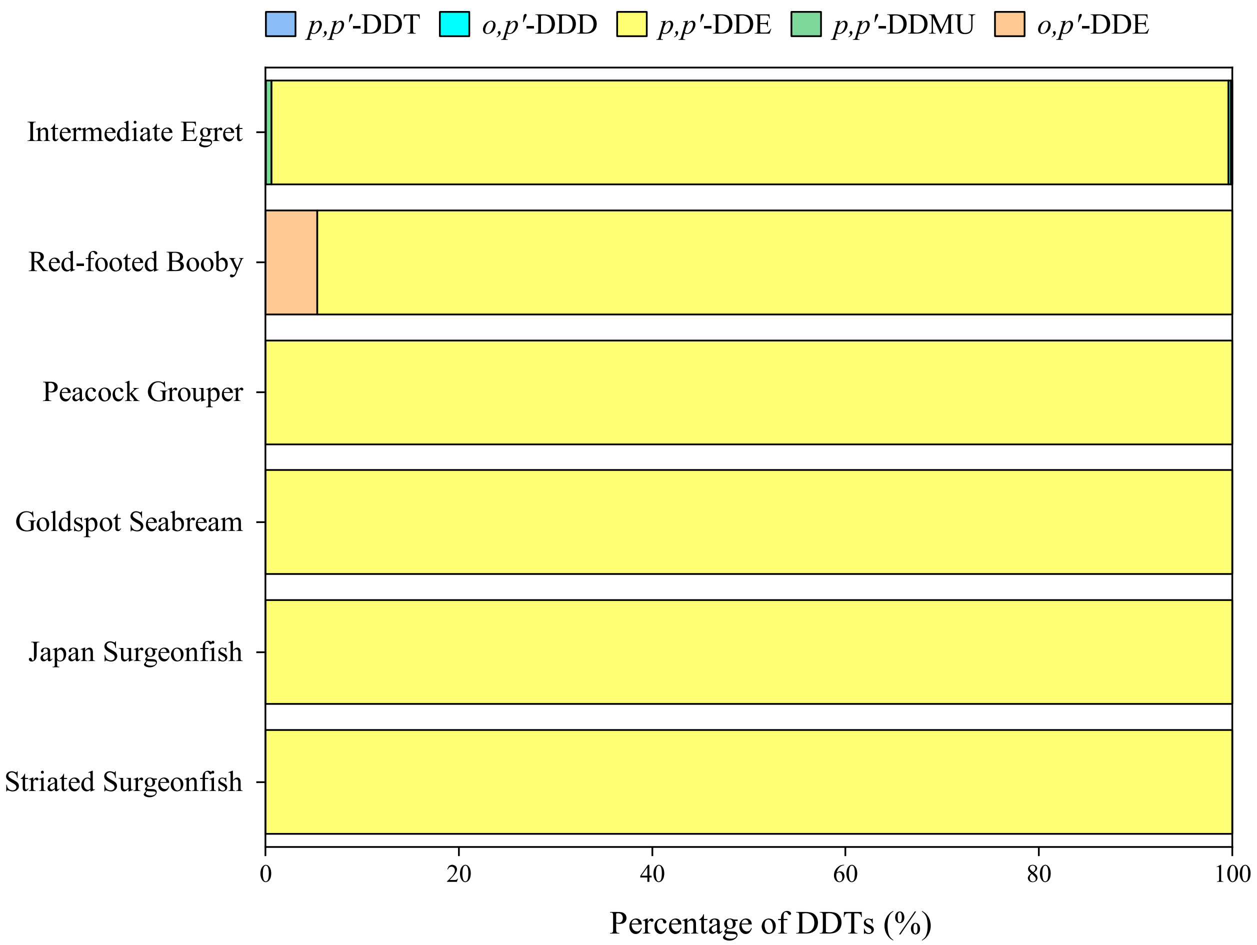


**Figure S2.** Composition profiles of DDTs in biota from Qilianyu Island.

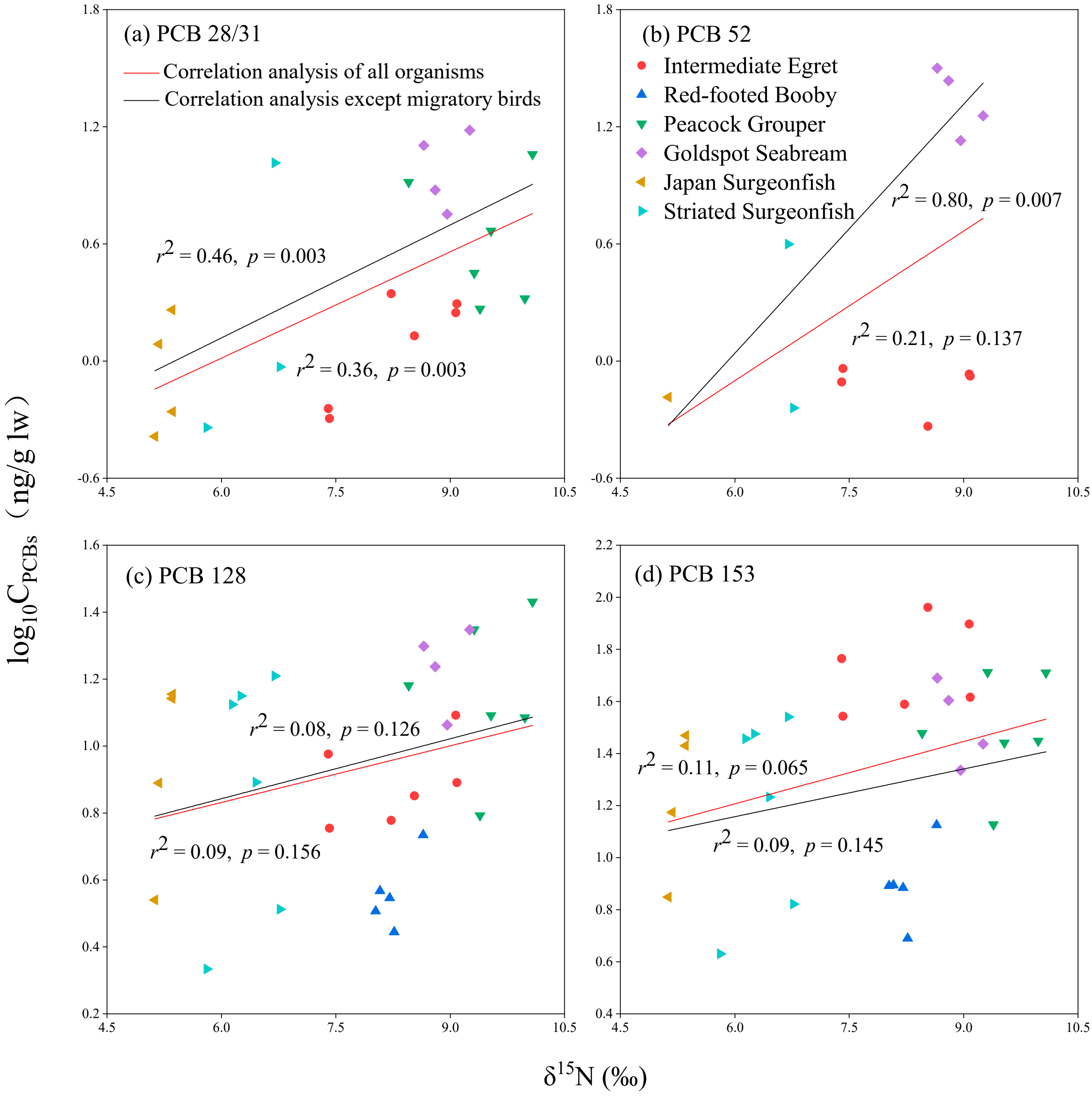


**Figure S3.** Relationships between concentrations of PCB congeners and $\delta^{15}N$ in biota species from Qilianyu Island.

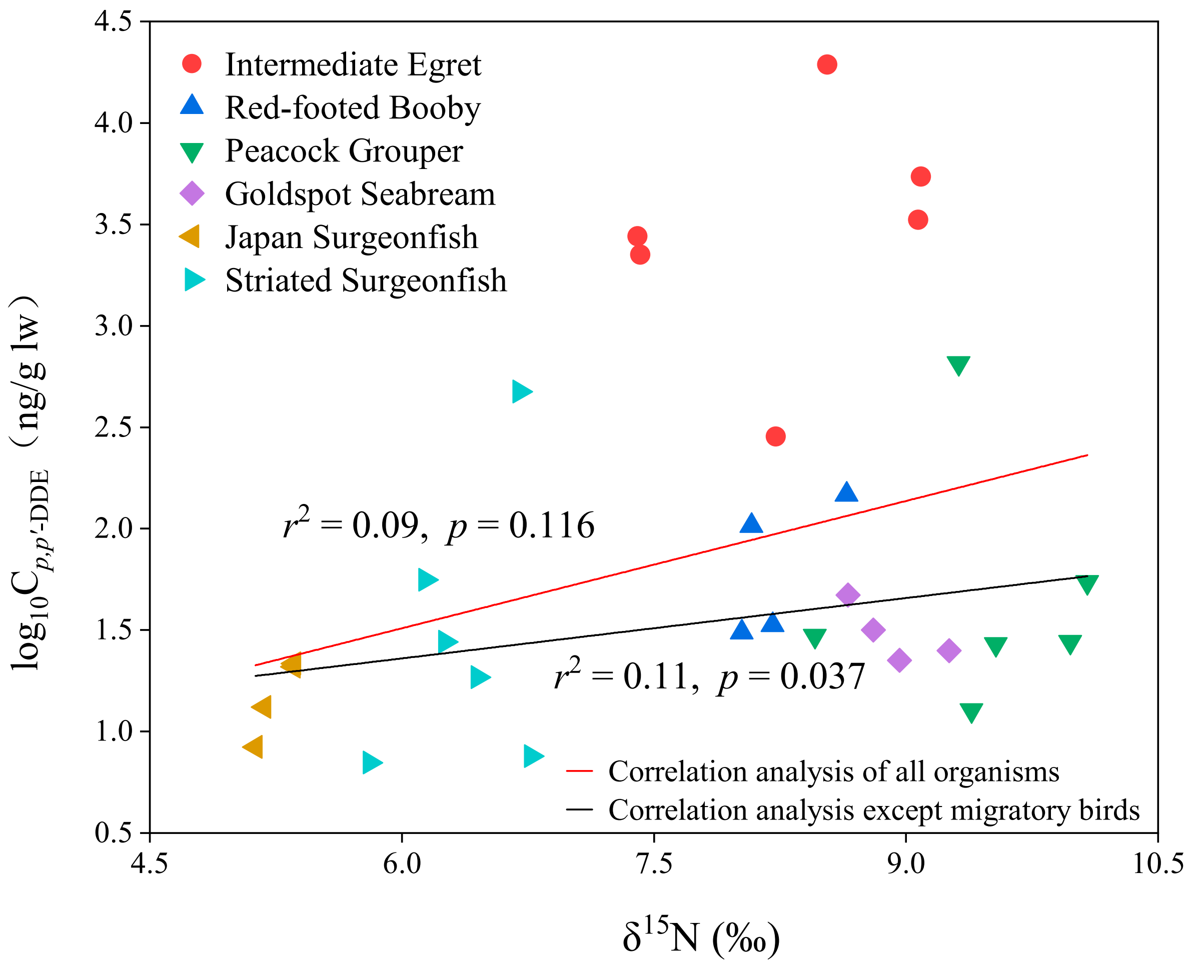


**Figure S4.** Relationships between concentrations of *p,p'*-DDE and $\delta^{15}N$ in biota species from Qilianyu Island.
